# PheneBank: a literature-based database of phenotypes

**DOI:** 10.1093/bioinformatics/btab740

**Published:** 2021-11-12

**Authors:** Mohammad Taher Pilehvar, Adam Bernard, Damian Smedley, Nigel Collier

**Affiliations:** Language Technology Lab, Department of Theoretical and Applied Linguistics, University of Cambridge, Cambridge, UK; The William Harvey Research Institute, Queen Mary University of London, London, UK; The William Harvey Research Institute, Queen Mary University of London, London, UK; Language Technology Lab, Department of Theoretical and Applied Linguistics, University of Cambridge, Cambridge, UK

## Abstract

**Motivation:**

Significant effort has been spent by curators to create coding systems for phenotypes such as the Human Phenotype Ontology, as well as disease–phenotype annotations. We aim to support the discovery of literature-based phenotypes and integrate them into the knowledge discovery process.

**Results:**

PheneBank is a Web-portal for retrieving human phenotype–disease associations that have been text-mined from the whole of Medline. Our approach exploits state-of-the-art machine learning for concept identification by utilizing an expert annotated rare disease corpus from the PMC Text Mining subset. Evaluation of the system for entities is conducted on a gold-standard corpus of rare disease sentences and for associations against the Monarch initiative data.

**Availability and implementation:**

The PheneBank Web-portal freely available at http://www.phenebank.org. Annotated Medline data is available from Zenodo at DOI: 10.5281/zenodo.1408800. Semantic annotation software is freely available for non-commercial use at GitHub: https://github.com/pilehvar/phenebank.

**Supplementary information:**

Supplementary data are available at *Bioinformatics* online.

## 1 Introduction

We contribute to the goal of understanding and curating human diseases by developing a high throughput Natural Language Processing (NLP) system that identifies phenotype and disease mentions in the scientific literature and links them to concept unique identifiers (CUIs) in biomedical ontologies. Integration of meaning between literature and concepts is an important task that has traditionally been accomplished using manually designed rules. In this work, we apply a BiLSTM-CRF neural network to go beyond straightforward lexico-orthographic variations such as *carotid arteries* and *Carotid artery*. Additional challenges of matching text strings to concept labels include (i) minimal lexical overlap between synonyms such as *reduced serum calcium concentration* and *hypocalcaemia*, (ii) polysemous relations such as between *digit* and *proximal phalanges*, (iii) partial matches such as *normal hearing sensitivity* and *hearing test normal* and (iv) complex compositionality relations such as *right-sided colorectal cancer* matching to a relation between *right* and *colorectal cancer* in SNOMED CT. Neural network approaches have recently been used in concept identification systems such as PubTator Central, although to the best of our knowledge PheneBank is the first to perform concept identification of phenotypic abnormalities directly to 13K Human Phenotype Ontology (HPO) terms (Köhler *et al.*, 2017). PheneBank brings together (i) API access to a state-of-the-art neural network model trained on complex sentences from full text articles for identifying concepts. The model exploits latent semantic representations (embeddings) to infer text-to-concept mappings in eight ontologies that would often not be apparent to conventional string matching approaches; (ii) text-level recognition of phenotype–disease associations calibrated against known biological relations provided by the Monarch Initiative using HPO-Mondo mappings; (iii) text search of all Medline abstracts incorporating PheneBank concepts; (iv) fully annotated 18.5M Medline abstracts accessed and the Europe PMC Annotations API (https://europepmc.org/AnnotationsApi).

When constructing a concept identification model, a major bottleneck is the lack of an openly available gold standard for evaluation. To address this issue, we make available the PheneBank Corpus consisting of 1.5K expert-annotated paragraphs selected from the PMC Text Mining (PMC-TM) subset (http://demo.phenebank.org/static/phenebank-data.zip). We believe this corpus offers advantages over Medline sentences due to the more complex linguistic structures they represent.

## 2 Materials and methods

**Fig. 1.**
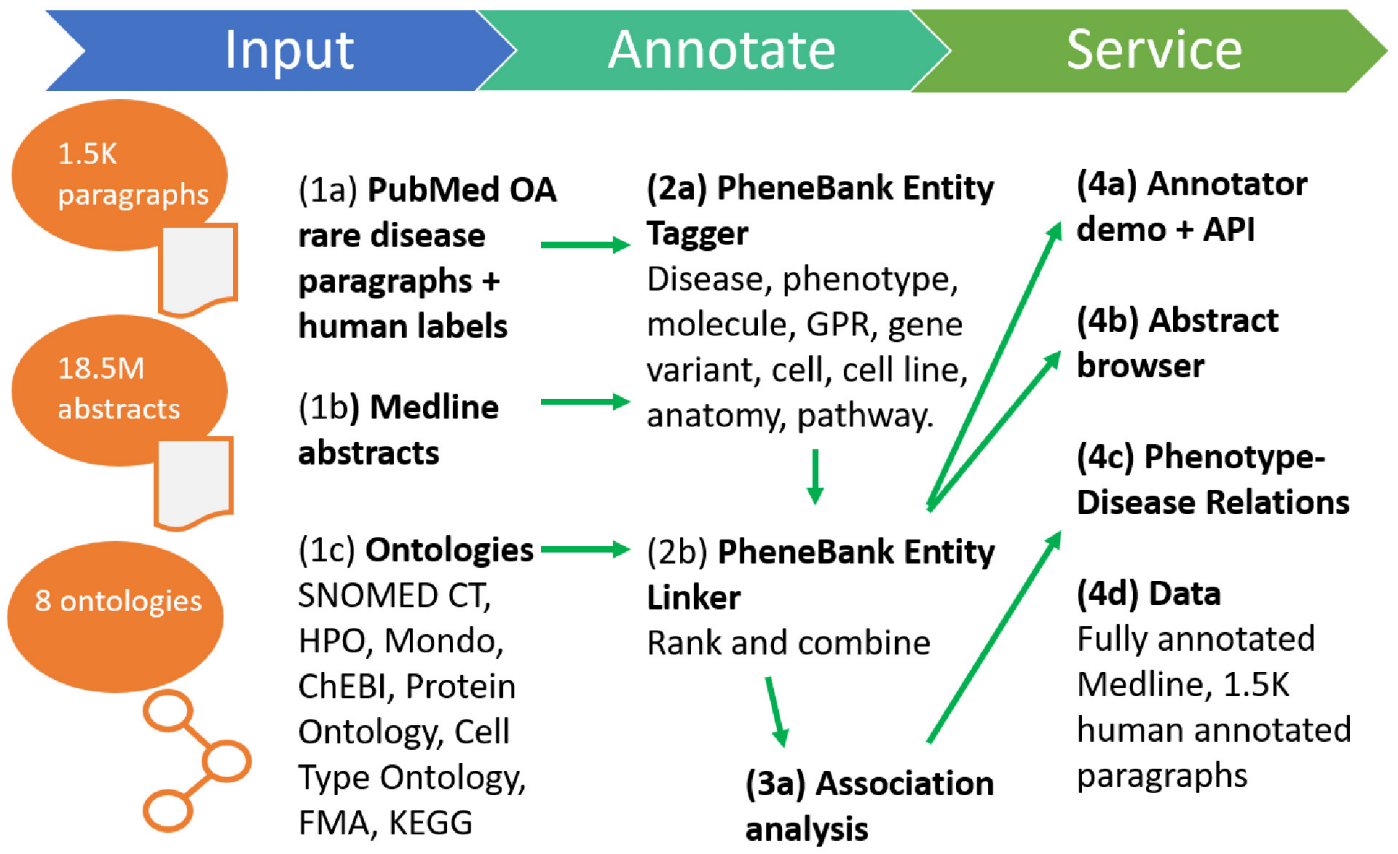
Overview of the PheneBank system

Figure  1 shows a high-level view of the information flow through the PheneBank system. End user services are deployed on a Linux server using Apache:

**Demo.** This service enables users to submit texts and receive NER annotations from PheneBank, e.g. Phenotype, Cell, Gene variant. We leverage a BiLSTM network for tagging entity mentions that exploits the desirable properties of the conventional Conditional Random Fields approach, such as sensitiveness to neighbouring context. The model implementation is based on anaGo (https://github.com/Hironsan/anago).

**Browser.** This service provides a search engine for the retrieval of automatically annotated content from 24M MEDLINE abstracts. Abstracts are annotated using the Named Entity Recognition (NER) module with all entities mapped (if possible) to a concept in one of the five major ontologies: SNOMED, HPO, MeSH, PRO and FMA. Users enter a query and retrieve all the relevant articles. System confidence is shown by colour intensity and concept details are shown by clicking on the corresponding entry. Mapping entities to concepts is carried out by unifying ontology and text entities based on lexical semantic spaces.

**Relations.** This service provides an interface to view the pre-computed disease–phenotype associations. Users can enter a disease name and check for the associated phenotypes and vice versa. 

## 3 Performance evaluation

**Entity tagging.** Training and evaluating NER taggers relies greatly on the availability of human-annotated data. To our knowledge, the *Gold Standard Corpora* (Groza *et al.*, 2015, GSC) is the only phenotype-tagged dataset. A major contribution of our work is the release of a large high-quality dataset tagged with nine classes of entities, including phenotypes. Supplementary Tables S1 and S2 show the NER tagging performance of our model and four other standard NER taggers on both the GSC and PheneBank datasets. Thanks to its usage of recurrent sequence encoders, our model greatly outperforms other systems (F1 0.69 on GSC versus 0.65 and F1 0.58 on PheneBank versus 0.36).

**Entity linking.**  Supplementary Table S3 shows the results for the quality of entity linking. The objective here is to map entities to corresponding concepts in HPO. For comparison, we show results for the NCBO Annotator and a string-based baseline which considers edit distance. Thanks to its usage of semantic composition of representations, PhenBank is able to improve on both conventional approaches (F1 0.78 versus 0.61 and 0.55).

**Phenotype–disease** **associations.** We employ a co-occurrence model where we assume that if a Disease and a Phenotype co-occur in a Medline abstract then there is a manifestation relationship between them. Given the volume of potential tuples, we compared a variety of statistical association measures to assess whether any are likely to represent a true biological relationship. We evaluated the Fisher Exact Test, the Dice coefficient and pointwise mutual information (PMI) ranking tuples from most significant to least significant. Then, we examined which metric best corresponded to the known tuples available from the curated associations available in the Monarch Initiative (https://monarchinitiative.org/). A good metric will tend to have high rankings for known tuples. Results (Data available at DOI: 10.5281/zenodo.142283.) showed that the Fisher Exact Test is clearly the best-performing of the three methods. Given that the Fisher Exact Test yields *P*-values, we then applied the Benjamini–Hochberg procedure to our dataset to find the cutoff for a false discovery rate of 1%; this occurs at *P* = 0.0025, after 1.8M tuples.

## 4 Conclusion

PheneBank is a database of phenotypes and their associations mined from the literature using machine learning techniques. We anticipate that the database will be useful in supporting biocuration and exploring phenotype-based similarity between diseases and patients as well as downstream text mining applications.

## Supplementary Material

btab740_supplementary_data
