# Supplementary material for: PheneBank: a literature-based database of phenotypes
Source: Bioinformatics. 2021 Nov 12;38(4):1179–80. doi: 10.1093/bioinformatics/btab740 (PMC8796364; doi:10.1093/bioinformatics/btab740)
Supplement: btab740_supplementary_data [file btab740_supplementary_data.pdf]

1 Supplementary Information

Table S1. **Phenotype tagging performance on the GSC dataset.**

System	Precision	Recall	F1
PheneBank BiLSTM-CRF	0.69	0.69	**0.69**
IHP (Lobo *et al.*, 2017)	0.56	0.79	0.65
OBO Annotator (Taboada *et al.*, 2014)	0.69	0.44	0.54
Bio-LarK CR (Groza *et al.*, 2015)	0.65	0.49	0.56
NCBO Annotator (Shah *et al.*, 2009)	0.54	0.39	0.45

The NCBO Annotator is based on the ontologies available in BioPortal (`https://bioportal.bioontology.org/`), the largest repository of biomedical ontologies. The OBO Annotator is a semantic Natural Language Processing tool capable of combining any number of OBO ontologies from the OBO foundry to identify their terms in a given text. Bio-LarK CR (Groza *et al.*, 2015) is an HPO concept recognition tool which defines a set of manually crafted pattern matching rules that enable capturing conjunctive terms. IHP (Lobo *et al.*, 2017) is an NER system tuned for recognizing phenotypic entities in unstructured texts. The system is based on Stanford CoreNLP (Manning *et al.*, 2014) for text preprocessing and Conditional Random Fields (CRF) for named entity recognition (NER). The CRF model leverages a rich set of features including linguistic, lexical, morphologic, orthographic, lexical, and context features. The system also benefits from a validation step that can filter incorrect annotations based on a set of manually crafted rules, such as the negative connotation analysis. We report results provided by Lobo *et al.* (2017): NCBO API (`http://data.bioontology.org/documentation`) targeted towards the HPO, the HPO-specific version of OBO Annotator available. linked to lexicons such as HPO.

Table S2. **Phenotype tagging performance on the PheneBank dataset.** We experimented with two settings: (1) phrases are regarded as whole units; partially tagging the phrases would not count towards correct results; (2) phrases are regarded as multiple disjoint entities; tagging any of the words counts toward overall performance.

	System	Precision	Recall	F1
Setting 1	BiLSTM-CRF	0.59	0.57	**0.58**
	IHP (Lobo *et al.*, 2017)	0.27	0.55	0.36
Setting 2	BiLSTM-CRF	0.78	0.79	**0.79**
	IHP (Lobo *et al.*, 2017)	0.49	0.58	0.53

Table S3. **Results for grounding to HPO entities.**

System	Accuracy
PheneBank - Semantic grounding	**0.78**
NCBO Annotator Shah *et al.* (2009)	0.61
Exact match baseline	0.55

References

Groza, T., Köhler, S., Doelken, S. C., Collier, N., Oellrich, A., Smedley, D., Couto, F. M., Baynam, G., Zankl, A., and Robinson, P. N. (2015). Automatic concept recognition using the human phenotype ontology reference and test suite corpora. volume 2015. bav005.

Köhler, S., Vasilevsky, N. A., Engelstad, M., Foster, E., McMurry, J., Aymé, S., Baynam, G., Bello, S. M., Boerkoel, C. F., Boycott, K. M., *et al.* (2016). The human phenotype ontology in 2017. *Nucleic acids research*, **45**(D1), D865–D876.

Lobo, M., Lamurias, A., and Couto, F. M. (2017). Identifying human phenotype terms by combining machine learning and validation rules. *BioMed Research International*, **2017**.

Manning, C. D., Surdeanu, M., Bauer, J., Finkel, J., Bethard, S. J., and McClosky, D. (2014). The Stanford CoreNLP natural language processing toolkit. In *ACL System Demonstrations*, pages 55–60.

Shah, N. H., Bhatia, N., Jonquet, C., Rubin, D., Chiang, A. P., and Musen, M. A. (2009). Comparison of concept recognizers for building the open biomedical annotator. *BMC Bioinformatics*, **10**(Suppl 9), S14.

Taboada, M., RodrÃguez, H., MartÃnez, D., Pardo, M., and Sobrido, M. J. (2014). Automated semantic annotation of rare disease cases: a case study. *Database*, **2014**.
